# A Rare Case of Trichilemmal Carcinoma of the Scalp

**DOI:** 10.7759/cureus.61807

**Published:** 2024-06-06

**Authors:** Raymart Macasaet, FNU Arty, Janelle du Toit, Sai Rakshith Gaddameedi, Shazia M Shah

**Affiliations:** 1 Internal Medicine, Monmouth Medical Center, Long Branch, USA; 2 Internal Medicine, Rutgers Health/Monmouth Medical Center, Long Branch, USA

**Keywords:** skin mass, cancer, trichilemmal cyst, scalp tumor, trichilemmal carcinoma

## Abstract

Trichilemmal carcinoma (TC) is a rare, low-grade, malignant adnexal tumor. It is usually less than 3 cm long and arises from the external root sheath of the hair follicle, most commonly in sun-exposed areas of the body. The treatment of choice is wide local excision with tumor-free margins. We present an 88-year-old male patient who presented with an incidental large, dry, fumigating mass on his scalp for a one-year duration requiring surgical excision. The mass, initially thought to be a benign sebaceous cyst, was a 12-cm trichilemmal carcinoma diagnosed based on the histopathologic features of the mass. The specimen was composed of keratinaceous material and necrotic debris. The viable tumor was consistent with atypical squamous proliferation. The mass was fully excised down to the scalp on the first encounter, leaving no further tissue to excise. The patient’s scalp site remained clean and without bleeding or recurrence. Currently, there is an increasing incidence of trichilemmal carcinoma. The pathophysiology of this disease is still unclear. The radiation from the sun is one of the factors that causes the growth of the lesions due to its location and distribution. Trichilemmal cysts can also transform into malignant trichilemmal carcinomas due to the p53 deletion. TC has a non-aggressive course despite its aggressive histology. The prognosis is generally good as it has low metastatic potential, like cutaneous squamous cell carcinoma. However, TC with metastasis has a poor prognosis, and there is no consensus yet on treatment. For non-metastatic TC, simple surgical excision with adequate (0.5-1 cm) margins is an effective treatment. Different studies use different margins, and there is no consensus on the measurement for margin excision. Regular follow-up is recommended, but further studies regarding follow-up schedules are needed. Furthermore, despite the common use of chemotherapy in cases of malignant TC, only a limited number of studies have explored this treatment approach. Given the increasing incidence of the disease, we highly recommend more research to address this knowledge gap.

## Introduction

Trichilemmal carcinoma (TC) is a rare, low-grade, malignant adnexal tumor. It is usually less than 3 cm and arises from the external root sheath of the hair follicle, most commonly in sun-exposed areas of the body [1]. The scalp, neck, and face are the most common sites of involvement. The trunk, back, buttocks, and vulva are occasionally also affected [2]. It was initially identified as tricoleptocarcinoma in 1968 and has an incidence rate of 0.05%. It commonly presents in the elderly population, mainly at the age of 70, with male predominance. The histological similarities to squamous cell carcinoma and the clinical similarity to other benign skin conditions result in delayed diagnosis. Hence, high clinical suspicion and accurate histopathological evaluation are needed for diagnosis confirmation [3]. The absence of a granular layer between the stratum spinosum and stratum corneum in a tumor exhibiting trichilemmal keratinization is the primary histological characteristic that distinguishes a TC from other types of tumors [4]. The treatment of choice is wide local excision with tumor-free margins.

## Case presentation

**Figure 1:**
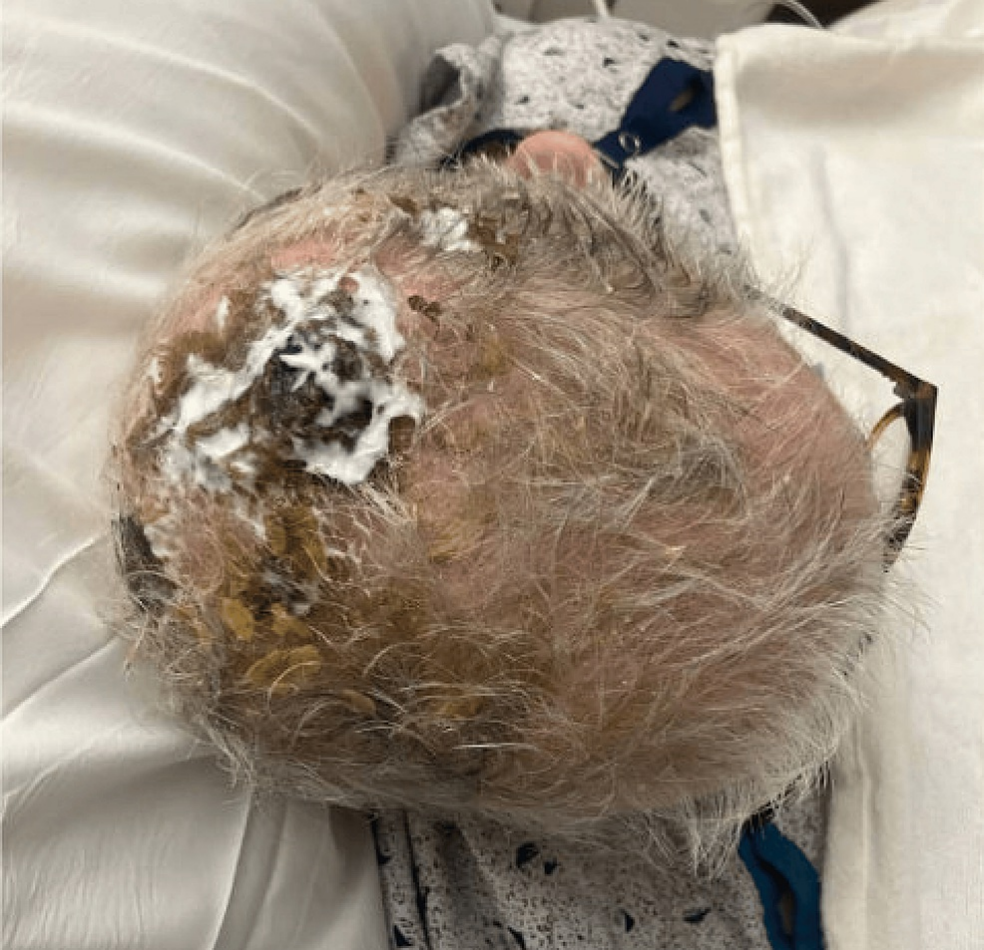
Posterior scalp wound

The patient is an 88-year-old male with a past medical history of hypertension, heart failure with a mildly reduced ejection fraction, post-automated implantable cardioverter-defibrillator insertion, bioprosthetic aortic valve replacement, gout, depression, benign prostatic hyperplasia, and obstructive uropathy with a chronic indwelling Foley catheter, which was found to have an incidental scalp mass. The patient’s family sought medical evaluation for the patient due to persistent mass growth that had been ongoing for one year prior. There were no complaints of associated scalp pruritus, bleeding, or discharge. The examination was significant for a large, dry, fumigating scalp mass without bleeding or drainage (Figure 1).

**Figure 2:**
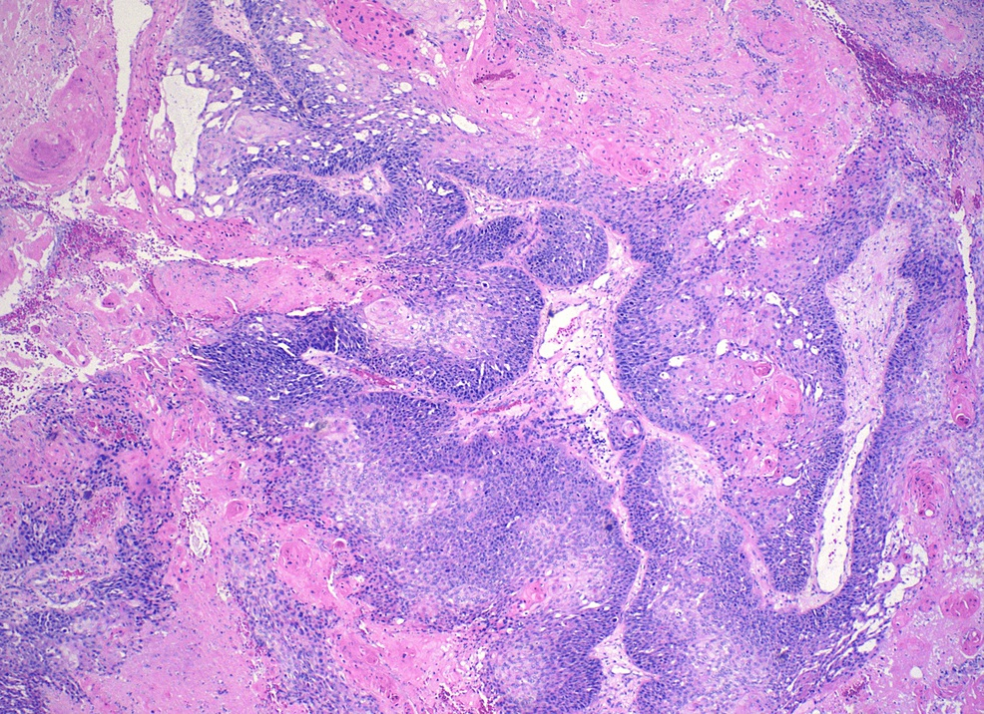
Abrupt trichilemmal keratinization and lack of granular layer

**Figure 3:**
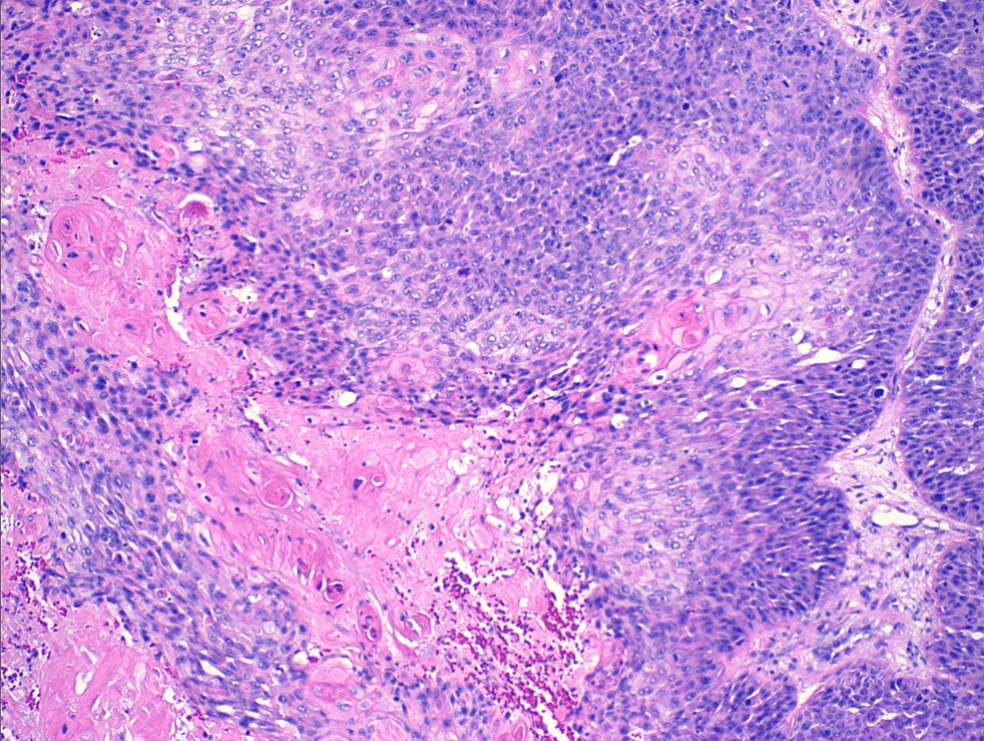
Abrupt trichilemmal keratinization and lack of granular layer (high power magnification)

**Figure 4:**
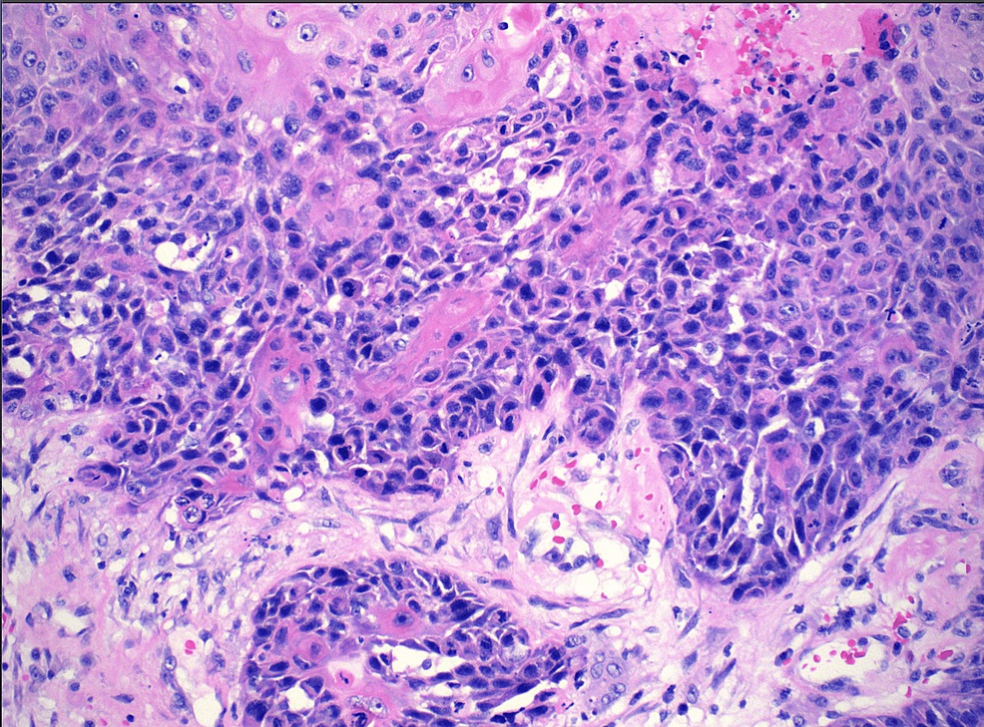
Irregular, polygonal, and hyperchromatic nuclei and marked pleomorphism

**Figure 5:**
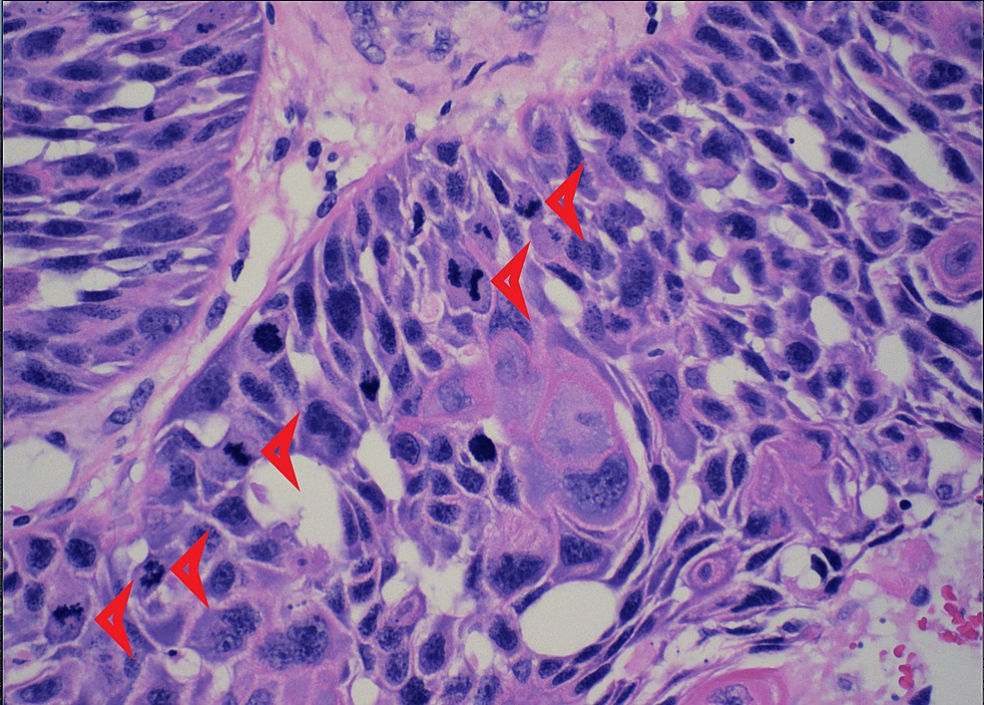
Abnormal mitosis (red arrowheads) with numerous inflammatory infiltrates were seen

A surgical excision revealed a scalp mass of 12 cm and was submitted for pathology evaluation. Initially, the mass was suspected to be a benign sebaceous cyst. Pending pathology results, the patient’s scalp site remained clean and was without bleeding or mass recurrence. The patient’s tissue pathology resulted in a positive for trichilemmal carcinoma. Histologically, the tissue illustrates abrupt trichilemmal keratinization and a lack of granular layer (Figures 2-3). Atypical squamous cells, which distinguish trichilemmal carcinoma from the benign variety, were noted (Figures 4-5). The specimen has features of irregular, polygonal, and hyperchromatic nuclei and marked pleomorphism (Figure 4). Figure 5 shows abnormal mitosis (red arrowheads) with numerous inflammatory infiltrates.

However, the majority of specimens were composed of keratinaceous material and necrotic debris. The viable tumor was consistent with atypical squamous proliferation with trichilemmal keratinization. Pathology was unable to define margins due to insufficient specimens for such. The patient was re-evaluated by surgery for another specimen. However, since the mass was fully excised down to the scalp on the first encounter, there was no further tissue to be removed. Given the rare diagnosis, this case was discussed with the pathologist in detail. The diagnosis was made based on the histopathologic features of the mass. Being an incidental finding, the medical team could not test for markers. It was decided by the medical and surgical team that chemotherapy and radiation were not needed because there was no evidence of metastasis and there was no tissue left to remove.

## Discussion

Trichilemmal carcinoma is a rare, malignant, cutaneous tumor that has a clinically indolent course [5,6]. There is an increasing incidence of this malignancy [7]. It grows from the outer root sheath of hair follicles and can be solitary or multiple [8]. It occurs in the elderly - men ages 60 to 80 years old and women ages over 80 - with no predilection to either sex [5]. The mean age of diagnosis is 70 years old [5]. It is usually seen in fair-skinned individuals in sun-exposed hair-bearing areas such as the scalp and posterior neck [6].

We report a case of an 88-year-old male who has trichilemmal carcinoma that clinically appeared as a large, dry, fumigating mass on the scalp, which was diagnosed by excision. It was initially thought to be a benign sebaceous cyst. Differential diagnoses for TC are squamous cell carcinoma, basal cell carcinoma, and keratoacanthoma, among others [9].

The pathophysiology of this disease is still unclear. The radiation from the sun is one of the factors that causes the growth of the lesions due to its location and distribution [10]. Trichilemmal cysts can also transform into malignant trichilemmal carcinomas due to p53 deletion [11,12].

TC has a non-aggressive course [13], despite its aggressive histology. The prognosis is generally good as it has low metastatic potential, similar to cutaneous squamous-cell carcinoma (SCC) [8,10]. However, TC with metastasis has a poor prognosis, and there is no consensus yet on treatment [8]. A study by Hayashi et al. used the CAV chemotherapy treatment (cisplatin, adriamycin, and vindesine), which is the regimen for highly advanced cases of SCC [14,15]. Yi et al. reported a case of a 60-year-old woman with malignant TC who underwent four cycles of cisplatin and cyclophosphamide treatment but only achieved a partial remission [16]. The patient eventually died.

For non-metastatic TC, simple surgical excision with an adequate (0.5-1 cm) margin is an effective treatment [8,12]. It is low-cost and safe [7,8]. Mohs micrographic surgery is also an option and has been used as a treatment in several case reports [17,18]. There are also patients with trichilemmal carcinoma treated with 5% topical Imiquimod cream [18,19]. The patient in this case report was treated with surgical excision, and there was no recurrence noted after two months. At present, there are no established follow-up guidelines for patients with TC after surgical excision. It is important to monitor patients to catch early recurrence and metastasis. In a 14-year study by Xu et al., they reported a median follow-up of 63.8 months in patients with trichilemmal carcinoma [20].

## Conclusions

Trichilemmal carcinoma has been increasing in incidence. It is a rare adnexal tumor of low metastatic potential that can safely be treated with simple excision. We report a rare case of non-metastatic trichilemmal carcinoma that was treated safely and effectively by simple excision. Different studies use different margins, and there is no consensus on the measurement for margin excision. The patient was followed up after two months, and regular follow-up is recommended. However, further studies regarding the follow-up schedule are needed. In addition, in cases of malignant TC, treatment with chemotherapy is commonly used, but there are only limited studies investigating this. The patient did not undergo chemotherapy and radiation as there was no evidence of metastasis, and the mass was fully excised. More research to address the knowledge gap in malignant TC management is highly recommended, given the increasing incidence of the disease.
